# Morphology and function in the Cambrian Burgess Shale megacheiran arthropod *Leanchoilia superlata* and the application of a descriptive matrix

**DOI:** 10.1186/1471-2148-12-162

**Published:** 2012-08-30

**Authors:** Joachim T Haug, Derek EG Briggs, Carolin Haug

**Affiliations:** 1 Zoological Institute and Museum, Department of Cytology and Evolutionary Biology, University of Greifswald, Soldmannstr. 23, 17487, Greifswald, Germany; 2 Department of Geology and Geophysics, Yale University, PO Box 208109, New Haven, CT, 06511, USA; 3 Yale Peabody Museum of Natural History, Yale University, New Haven, CT, 06520, USA

**Keywords:** Megacheira, Great-appendage arthropods, Chelicerata *sensu lato*, Descriptive matrix, Active predator

## Abstract

**Background:**

*Leanchoilia superlata* is one of the best known arthropods from the middle Cambrian Burgess Shale of British Columbia. Here we re-describe the morphology of *L. superlata* and discuss its possible autecology. The re-description follows a standardized scheme, the descriptive matrix approach, designed to provide a template for descriptions of other megacheiran species.

**Results:**

Our findings differ in several respects from previous interpretations. Examples include a more slender body; a possible hypostome; a small specialised second appendage, bringing the number of pairs of head appendages to four; a further sub-division of the great appendage, making it more similar to that of other megacheirans; and a complex joint of the exopod reflecting the arthropod’s swimming capabilities.

**Conclusions:**

Different aspects of the morphology, for example, the morphology of the great appendage and the presence of a basipod with strong median armature on the biramous appendages indicate that *L. superlata* was an active and agile necto-benthic predator (not a scavenger or deposit feeder as previously interpreted).

## Background

The description of species is fundamental to the science of zoology, including taxonomy, phylogenetic systematics, functional morphology and ultimately evolutionary biology and ecology. But living organisms can never be completely described at every level of detail down to cellular morphology. It is easier to describe fossil species entirely, but only because much less detail is available. However, many descriptions of fossils are inadequate to allow them to be used directly to prepare cladistic matrices for phylogenetic analyses. This is usually because authors concentrate on morphological features that differentiate new species from those previously described. Thus, the focus is on structures that are unique even though cladistic matrices require structures that are shared with other species. As a consequence, morphological details in many phylogenetic matrices have to be (re-)interpreted, often without the benefit of a comprehensive description. While every character and character state should be carefully reviewed prior to analysis [1-4], it is also essential to provide explicit explanations of the basis for coding.

The so-called short great-appendage arthropods have been incorporated into many recent arthropod phylogenies. Short great-appendage arthropods, which, together with Chelicerata *sensu stricto*, form Megacheira [5], have only been reported from Cambrian strata. The characteristic short great appendage is the first appendage on the head. In their redescription of *Yohoia tenuis* Walcott, 1912, one of these arthropods from the middle Cambrian Burgess Shale biota of British Columbia, Haug JT et al. [5] noted that previous phylogenetic analyses of the short great-appendage arthropods included incomplete and/or controversial codings. The present paper represents a further step in addressing this problem: a redescription of *Leanchoilia superlata* Walcott, 1912, one of the most abundant short great-appendage arthropods.

*Leanchoilia superlata* is a relatively common megacheiran arthropod (more than a thousand specimens) from the Burgess Shale. García-Bellido and Collins [6] redescribed it and resurrected the species *L. persephone* Simonetta, 1970, which had been synonymized with *L. superlata* by Bruton and Whittington [7], a view supported by Briggs and Robison [8]. García-Bellido and Collins [6] also indicated that the material synonymized with *L. superlata* by Bruton and Whittington [7] might include other valid species. One of these, *L. protogonia* Simonetta, 1970, was subsequently resurrected by Briggs et al. [9].

Our reconsideration of *L. superlata* here is prompted by a number of factors. García-Bellido and Collins [6] focused on characters useful for distinguishing *L. superlata* from other species of *Leanchoilia* present in the Burgess Shale. Here we pay equal attention to other morphological details that might be important in determining the phylogenetic position or autecology of *Leanchoilia*. New information on *Leanchoilia illecebrosa* (Hou, 1987) from the lower Cambrian Chengjiang lagerstätte of China, published by Liu et al. [10], was not available to García-Bellido and Collins [6]. A direct comparison of these two species is desirable as *L. illecebrosa* has been identified as the sister species of *L. superlata*[4] and the morphology of one might illuminate that of the other. García-Bellido and Collins [6] discussed the relationships of *Leanchoilia superlata* in the context of the arachnomorph concept [11,12]. We reconsider its affinities in the light of increasing evidence that megacheirans are derivatives of the evolutionary lineage leading to modern chelicerates, representing a monophyletic group together with Chelicerata *sensu stricto*[5,13,14].

The redescription of *Leanchoilia superlata* here provides a template for future investigations of other megacheirans; comparable details of other taxa would allow more robust phylogenetic and evolutionary interpretations.

## Methods

### Material

Specimens in the collections of the Royal Ontario Museum, Toronto (ROM), previously identified as representatives of *Leanchoilia*, were inspected: a total of 1,253 slabs, very few with more than one specimen. These specimens were the basis for the study by García-Bellido and Collins [6], who provided details of the material and geological setting. 170 specimens preserved sufficient detail to merit being photographed (see below). Only specimens attributed to *L. superlata* are discussed here. A comparison with other species of *Leanchoilia* will be the topic of a separate paper.

#### Documentation and image processing

All specimens were inspected dry with a Nikon SMZ 1500 stereomicroscope under cross-polarized light. Specimens were photographed dry under cross-polarized light. Only in very rare cases did wetting specimens and photographing them under reflected light (high angle) without polarizers provide better contrast. Images were taken with a Canon Rebel T3i camera, equipped with either an EF-S 18-55 mm lens or a MP-E 65 mm macro lens. Lighting was provided by a MeiKe FC 100 LED macro ring flash and additional fiber light sources. Images were often recorded as a stack at different focal planes, especially for close-ups. Stacks were fused with CombineZM or CombineZP. Overview images, in particular, were taken as several photographs in different x- and y-positions and then stitched together with Adobe Photoshop CS3 or Microsoft Image Composite Editor (for details on composite imaging see [15]). Images were color- and contrast-optimized in Adobe Photoshop CS3 or GIMP 3.0. Unsharp mask filters were applied in Adobe Photoshop CS3. The 3D computer model was reconstructed using Blender.

#### New terminology

**Figure 1:**
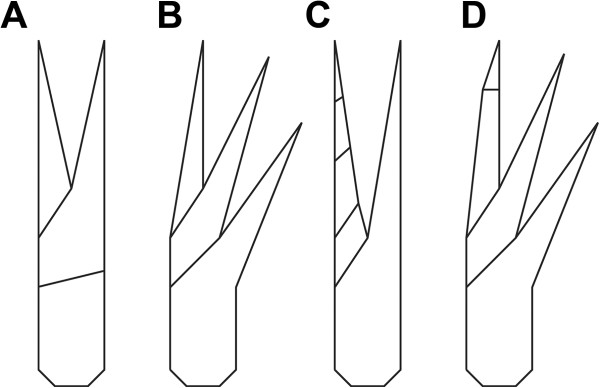
**Chela terminology.A**, “Normal” chela represented by a chelicera; **B**, Multi-chela: the second element is functionally a movable finger for the next proximal element and at the same time a fixed finger for the distal element, as in the megacheiran *Haikoucaris ercaiensis* Chen, Waloszek and Maas, 2004 [14]; **C**, Chela with a functional movable finger consisting of more than one element, as in the heteropteran *Tydides rufus* (Serville, 1831) [38]; **D**, Multi-chela with a distal chela with a functional movable finger consisting of two elements, as in the great appendage of *Leanchoilia superlata* Walcott, 1912.

The great appendage has been interpreted as being homologous to the chelicera of Chelicerata *sensu stricto* ([4,5,14] but see, for example, [16]). The distal two elements of a chelicera form a chela with a proximal fixed finger (digitus fixus) and a distal movable finger (digitus mobilis) (Figure 1A). The megacheiran great appendage comprises several functional chelae, in which an element forms a movable finger against the one proximal to it, while forming a fixed finger in relation to the one distal to it (Figure 1B). Thus the normal terminology for chelicerae is inadequate. We refer to this type of claw with more than two fingers as a 'multi-chela'. This term is applicable not only to the great appendage of megacheirans but also, for example, to the pedipalps of thelyphonid uropygids.

The movable finger in the great appendage is usually a single element, i.e., a spine arising from the next distal element, but it may include more than one. Such an arrangement occurs not only in *Leanchoilia*, but also, e.g., in different (sub-)chelate appendages in various crustaceans and insects (Figure 1C) and in the first appendage in *Amplectobelua*[17]. The different functional chelae of a multi-chela can be described independently: that in *Leanchoilia superlata*, for example, is composed of a proximal chela with a movable finger including a single element (the movable finger is the spine arising from the next distal element) and a distal chela where the movable finger includes two elements (Figure 1D).

#### Standardized descriptions

In order to allow direct comparison of structures in different megacheirans, we propose a standardized description. Clearly if an interesting structure is discovered in one species, it is necessary to check for its occurrence in others (Hennig’s principal of reciprocal illumination). Even if a structure were present in closely related species, it may not have been considered sufficiently important to warrant a mention. A morphological re-interpretation is usually triggered in this way - with the discovery of a new detail in a related species.

New discoveries and new interpretations often make it necessary to re-work species descriptions [2]. However, this process should not be restricted to supplementing or modifying data matrices, new interpretations of morphological characters should be properly explained and justified. This could be achieved by handling descriptions in a similar way to matrices, with a systematic treatment of character states and presence/absence information. Comparable approaches have been used in database projects like DELTA (DEscription Language for TAxonomy; [18]) although this program is neither readily accessible nor appropriate in its recent form.

Here we follow a simple 'descriptive matrix' approach, compiling the description in an xls.-file in OpenOffice with appropriate rows and columns (see Additional file 1). Following this approach will ensure that subsequently described species will be checked for all characters. When new characters come to light they can be added, but need to be checked on all species. The application of such an approach will result in a comparable set of descriptions that can be converted easily into a cladistic matrix (some characters may be dependent or reciprocal, and therefore omitted). Yet, the whole process must remain an iterative one: with the addition of each new species, new characters will be introduced, which then will have to be described in already named species. The descriptive matrix approach is established here for the short great-appendage arthropods, a small group of morphologically similar species. Expanding this approach to a larger group such as Megacheira will require a clear discussion of assumed character homologies. Even though such a process will be labor-intensive, the descriptive matrix provides a tool to make the enterprise transparent and comprehensible.

The descriptive matrix approach generates a data set that can be readily converted to a “plain language” description of the species (term from DELTA) which is easily compared to other descriptions using the same matrix. We will use the re-description of *Leanchoilia superlata* generated in this way here as a basis for preparing descriptions of other leanchoiliid species. Although it is straightforward to generate text directly from the matrix, we provide such a description here, but shortened to avoid repetitions.

## Results

### General remarks

The studied specimens of *Leanchoilia superlata* range in size from ca 24 mm to 70 mm. It was not possible to distinguish ontogenetic stages on the basis of measurements, nor did changes in morphology occur within this size range. Where structures vary in morphology, e.g., the number of setae on the exopods, such changes do not correlate with size and may reflect differences in preservation. Where the limitations of preservation resulted in uncertainties (e.g., in the posterior appendages) these are indicated with queries in the description (see below). For the purposes of reconstruction such missing information was inferred from the adjacent appendage(s) assuming serial similarity.

### Description

#### General form

**Figure 2:**
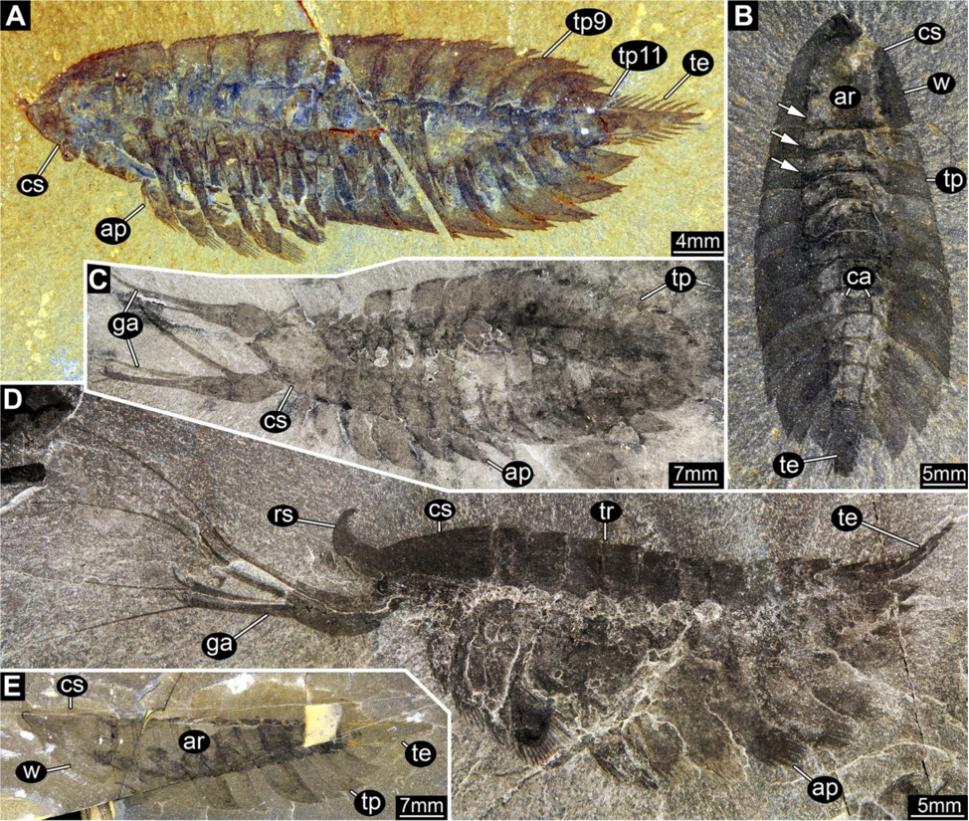
*** Leanchoilia superlata *Walcott, 1912, general body shape.A**, ROM 61896, ventral view with appendage preservation, note the serration of the cephalic (head) shield and tergo-pleurae; **B**, ROM 61891, dorsal view, showing the narrow axial region, arrows mark border between axial region and tergo-pleurae; **C**, ROM 61884, dorsal view with great appendages, image flipped horizontally; **D**, ROM 61882, lateral view of rather complete specimen in supposed life position, less well preserved area at the lateral margin of the axial region indicates the former horizontal position of the tergo-pleurae; **E**, ROM 61909, rather “translucent” preservation of the specimen emphasizes the slenderness of the central body region compared to the long tergo-pleurae, image flipped horizontally; abbreviations: ap = appendage; ar = axial region; ca = carinae; cs = cephalic shield; ga = great appendage; rs = rostrum; te = telson; tp = tergo-pleura; tr = trunk; w = wing.

Small arthropod with an elongate body differentiated into head, segmented trunk and non-somitic telson (Figure 2). Body with 16 segments comprising an ocular and 15 post-ocular appendage-bearing segments (Figure 2A, B). Ocular segment and post-ocular segments 1–4 incorporated into the head, their dorsal area contributing to the head shield. Post-ocular segments 5–15 (corresponding to trunk segments 1–11) forming tergites dorsally.

#### Head

**Figure 3:**
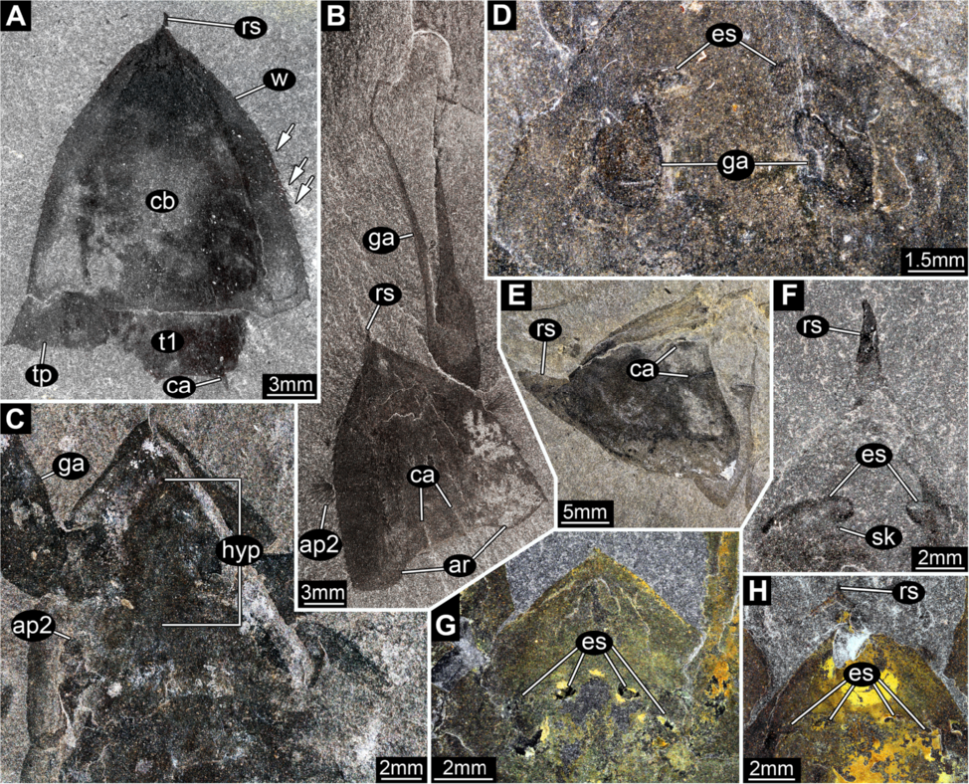
*** Leanchoilia superlata *Walcott, 1912, head region.A**, ROM 61869, dorsal view of head shield with rostrum and wings plus first trunk segment with carinae, note the marginal serration (arrows); **B**, ROM 61892, dorsal view of isolated head with great appendage and small second appendage, posterior shield area with carinae similar to those on the trunk tergites; **C**, ROM 61862, ventral view with hypostome, insertion areas of first and second appendage apparent; **D**, ROM 61894, dorsal view, a pair of eye structures and the insertions of the great appendages evident through the shield; **E**, ROM 61864, dorso-lateral view with dorsally curving rostrum, image flipped horizontally; **F**- **H**, Eye structures; **F**, ROM 61897, ventral view of eye structures with short stalks; **G**, ROM 61874; **H**, ROM 61887; additional abbreviations: es = eye structures; hyp = hypostome; sk = stalk; t = trunk segment.

**Figure 4:**
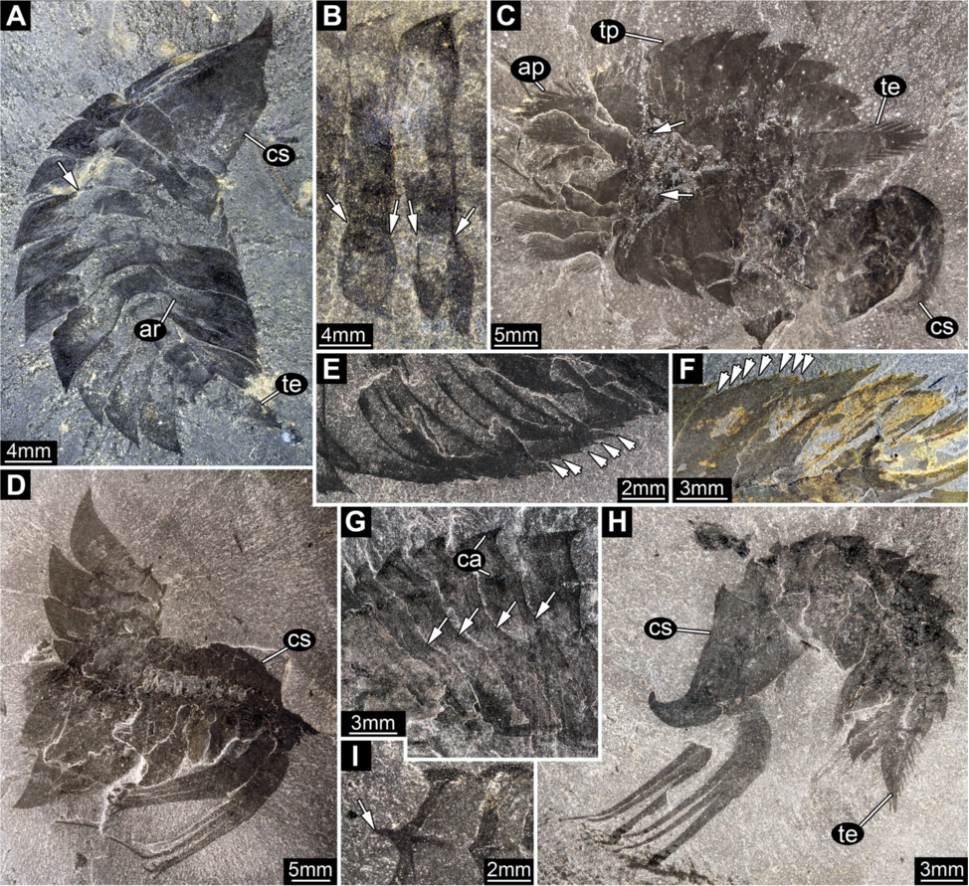
*** Leanchoilia superlata *Walcott, 1912, tergites and body flexure.A**, ROM 61870, dorsal view showing tripartite organisation of tergites, arrow marks border between axial region and tergo-pleurae, image flipped horizontally; **B**, ROM 61863, close up of tergites, arrows mark border between axial region and tergo-pleurae; **C**, ROM 61905, strongly dorsally flexed specimen, arrows indicate insertion areas of appendages; **D**, ROM 61875, lateral view of dorsally flexed specimen; **E**- **F**, Tergo-pleurae with serrate margin (arrows); **E**, ROM 61866; **F**, ROM 61871; **G**, ROM 61873, close up of tergites with carinae, arrows indicate border between axial region and tergo-pleurae; **H**, ROM 61906, strongly ventrally flexed specimen; **I**, ROM 61875, detail of carinae drawn out into posteriorly pointing spine (arrow); abbreviations as before.

Head shield with pronounced axial region and lateral ‘wings’ (Figure 3A), each wing region about one quarter of head shield width. Head shield shape almost triangular in dorsal view, about as long as wide at the posterior margin. Anterior rim of head shield drawn out into dorsally curved, hook-like rostrum (Figure 3E). Rim of head shield with up to 13 serrations on each side (Figures 2A, 3A). Posterior region of head shield (probably corresponding to post-ocular segment 4) resembles trunk tergites [cf. (Figure 4)] in the presence of a pair of carinae on the latero-dorsal area of the axis (Figure 3B, E). Carinae spine-like, pointing posteriorly.

#### Post-ocular segment 5: trunk segment 1

Length about one fifth that of the head shield. Total width slightly greater than that of the head shield. Width of axial region similar to that of the head shield. Tergo-pleural region on each side about one quarter entire width, almost straight, curving slightly posteriorly. Lateral rim with 7 to 8 serrations (Figures 2A-C, 4).

#### Post-ocular segment 6

Length as in preceding segment. Total width and width of axial region slightly greater than those of preceding segment. Tergo-pleural region as in previous segment. Lateral rim with about 7 serrations (Figures 2A-C, 4).

#### Post-ocular segment 7

Length as in preceding segment. Total width and width of axial region slightly greater than those of preceding segment. Tergo-pleural region on each side slightly more than one quarter entire width, curving slightly posteriorly at roughly 20°. Lateral rim with about 7 serrations (Figures 2A-C, 4).

#### Post-ocular segment 8

Length as in preceding segment. Total width and width of axial region slightly greater than those of preceding segment. Tergo-pleural region on each side slightly more than one quarter of entire width, curving posteriorly at roughly 30°. Lateral rim with 7 to 8 serrations (Figures 2A-C, 4).

#### Post-ocular segment 9

Length as in preceding segment. Total width slightly greater than that of preceding segment. Axial region slightly narrower than that of preceding segment. Tergo-pleural region on each side more than one quarter of entire width, curving posteriorly at roughly 40°. Lateral rim with 7 to 8 serrations (Figures 2A-C, 4).

#### Post-ocular segment 10

Length as in preceding segment. Total width slightly greater than that of preceding segment, largest of series. Axial region slightly narrower than that of preceding segment. Tergo-pleural region on each side slightly less than one third of entire width, curving posteriorly at roughly 40°. Lateral rim with 7 to 8 serrations (Figures 2A-C, 4).

#### Post-ocular segment 11

Length as in preceding segment. Total width slightly less than that of the preceding segment. Axial region slightly narrower than that of preceding segment. Tergo-pleural region on each side about one third of entire width, curving posteriorly at roughly 45°. Lateral rim with 7 to 8 serrations (Figures 2A-C, 4).

#### Post-ocular segment 12

Length as in preceding segment. Total width less than that of the preceding segment. Axial region slightly narrower than that of preceding segment. Tergo-pleural region on each side about one third of entire width, curving posteriorly at roughly 50°. Lateral rim with about 7 serrations (Figures 2A-C, 4).

#### Post-ocular segment 13

Length as in preceding segment. Total width less than that of the preceding segment. Axial region narrower than that of preceding segment. Tergo-pleural region on each side slightly less than one third of entire width, curving posteriorly at roughly 60°. Lateral rim with probably 7 serrations (Figures 2A-C, 4).

#### Post-ocular segment 14

Length about one sixth of the head shield. Total width about three-quarters of preceding segment. Axial region narrower than that of preceding segment. Tergo-pleural region on each side slightly less than one third of entire width, curving posteriorly at roughly 70°. Lateral rim with probably 7 serrations (Figures 2A-C, 4).

#### Post-ocular segment 15: trunk segment 11

Length as in preceding segment. Total width less than two thirds of the preceding segment. Axial region narrower than that of preceding segment. Tergo-pleural region on each side slightly less than one third of entire width, curving posteriorly at roughly 85°. Lateral rim with 6 to 7 serrations (Figures 2A-C, 4).

#### Telson

**Figure 5:**
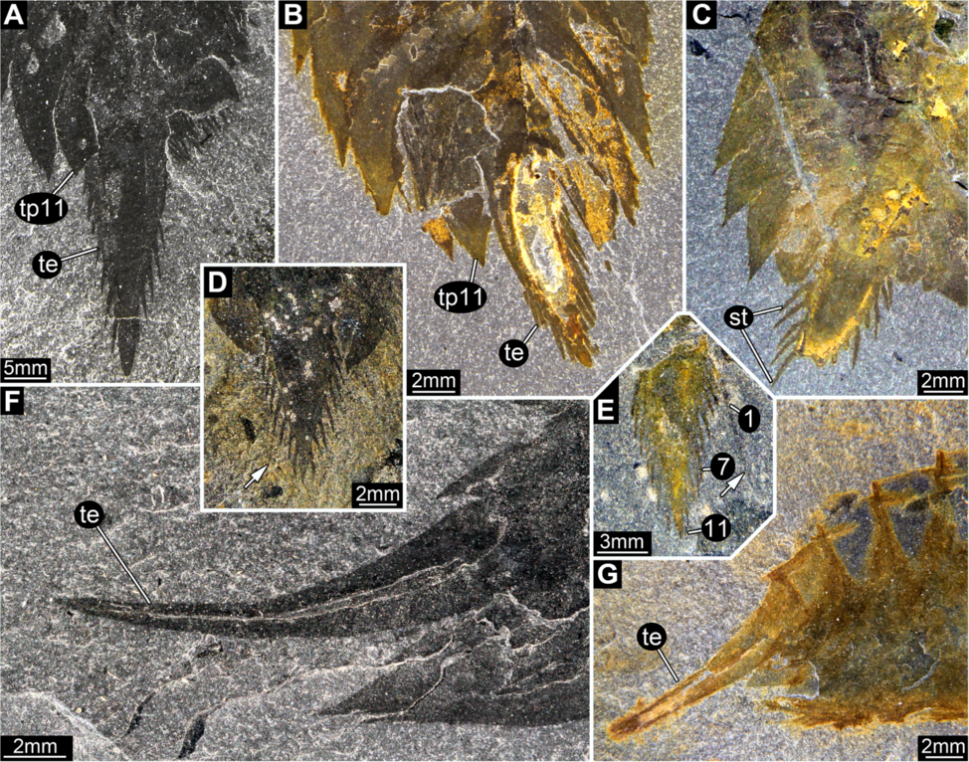
*** Leanchoilia superlata *Walcott, 1912, telson.A**- **E**, Dorso-ventral view; **A**, ROM 61880; **B**, ROM 61871; **C**, ROM 61874; **D**- **E**, Especially well preserved setae on the telson, arrows mark some very long setae; **D**, ROM 61868; **E**, ROM 61889, numbers indicate the number of setae; **F**- **G**, Lateral view; **F**, ROM 61878; **G**, ROM 61867; additional abbreviation: st = setae.

Telson elongate triangular in dorsal view (Figure 5A-E), scimitar-shaped in lateral view (Figure 5F-G), tip pointed. Setae around the entire distal and lateral margin, 11 on each side, uniform in size.

#### Eyes

Lateral eyes with short stalks arising from the antero-ventral region of the head (Figure 3D, F-H). Each eye consisting of two lobes.

#### Hypostome

Hypostome elongate drop-shaped, situated ventrally between the insertions of the eyes (anteriorly), appendage 1 (antero-laterally) and appendage 2 (postero-laterally) (Figure 3C). Presumably carrying the mouth opening posteriorly.

#### Appendage 1

**Figure 6:**
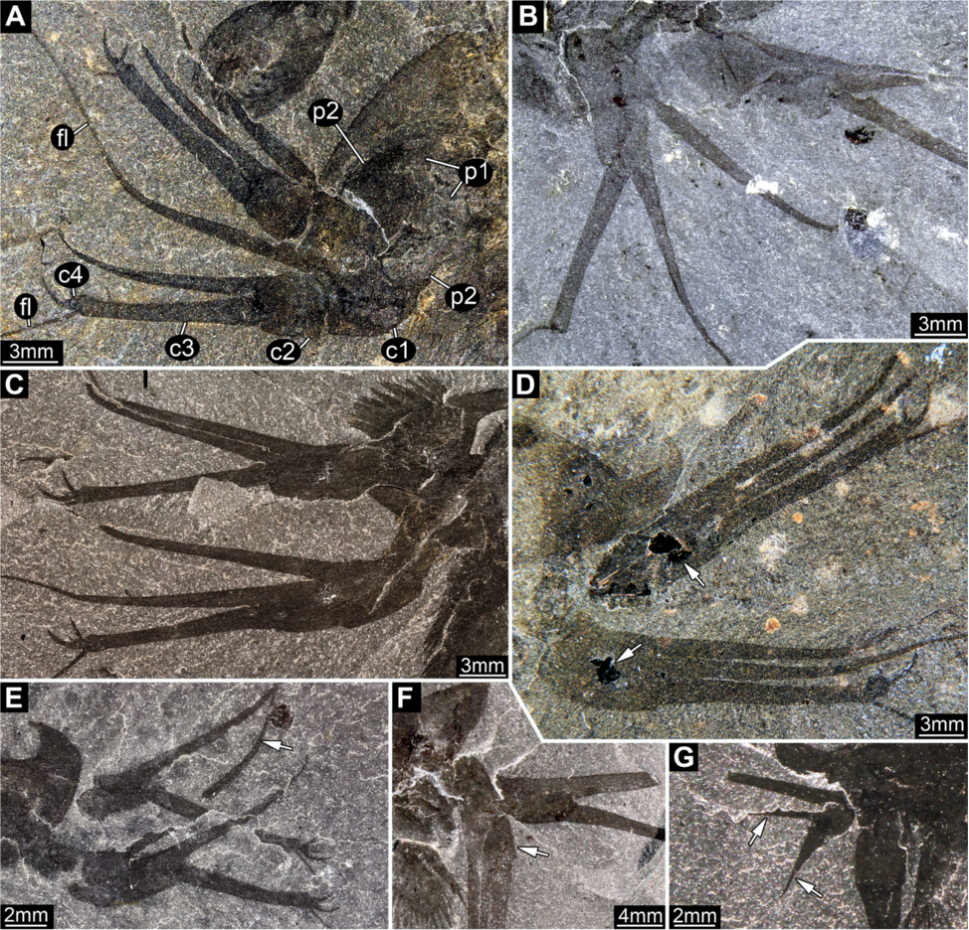
*** Leanchoilia superlata *Walcott, 1912, great appendage.A**, ROM 61888, fairly complete pair of great appendages, each appendage consisting of two peduncle elements and four claw elements, image flipped horizontally; **B**, ROM 61899, fingers of multi-chela spread out; **C**, ROM 61900, fingers of multi-chela more closed than in **B**; **D**, ROM 61890, fingers of multi-chela closed, arrows mark dark spots, which Bruton and Whittington [7] assumed to be excretory organs; **EG**, “Malformations” of great appendages; **E**, ROM 61898, flagella missing, finger bent more than usual (arrow); **F**, ROM 61878, partly disarticulated great appendage rotated postero-ventrally (arrow); **G**, ROM 61886, elements broken off (arrows); additional abbreviations: c = claw element; fl = flagellum; p = peduncle element.

**Figure 7:**
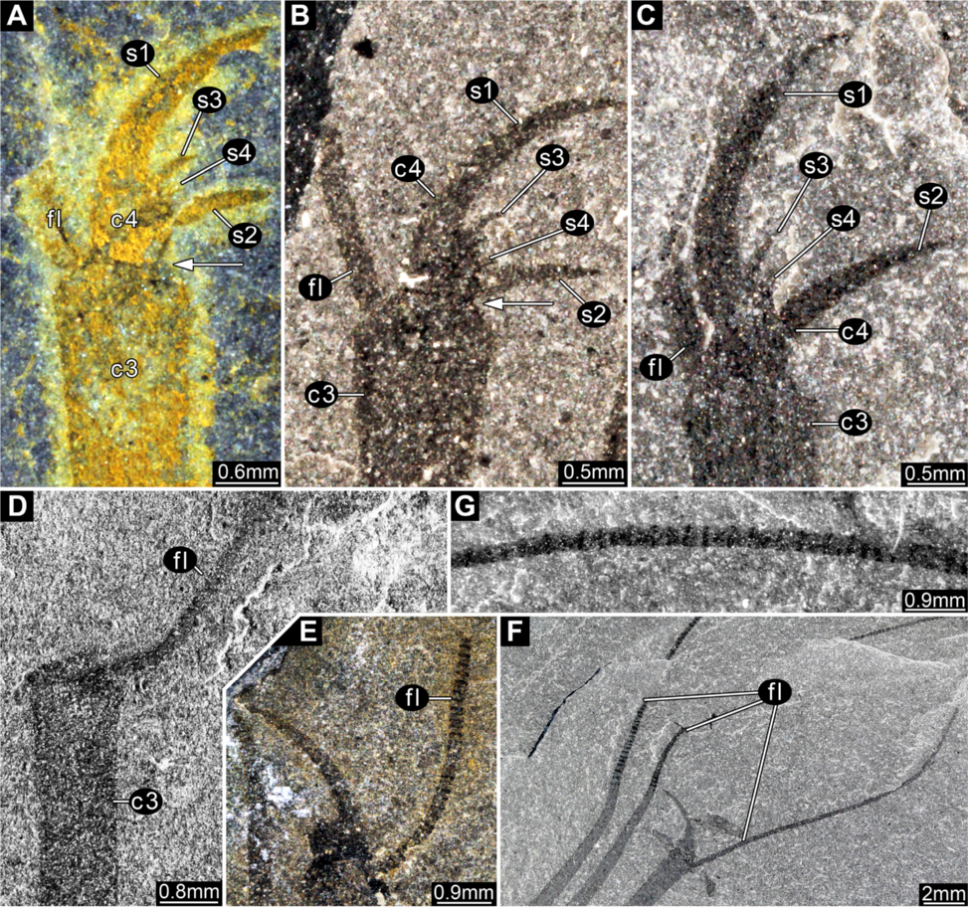
*** Leanchoilia superlata *Walcott, 1912, distal details of great appendage.A**- **C**, Distal armature, different spine positions indicate movability of claw element 4, arrows mark joint between claw elements 3 and 4; **A**, ROM 61885; **B**, ROM 61893; **C**, ROM 61900; D-G, Flagella; **D**, ROM 61899, claw element 4 disarticulated and missing; **E**, ROM 61876; **F**- **G**, ROM 61902; **G**, Detail of **F** with annulation of flagellum; additional abbreviation: s = spine.

Prehensile great appendage, differentiated into peduncle and claw (Figure 6A-D), separated by a hinge joint. Peduncle of two elements (Figure 6A) which are slightly shorter than wide (diameter). Claw a multi-chela with four elements (Figure 6A), the two distalmost forming a divided movable finger [cf. (Figure 1D)]. Claw element 1 short, as wide (diameter) as long, curved inward, with an elongate, medio-distal spine, directed distally, 3x as long as the basal part of the element (almost as long as the head shield). The diameter of the spine at its base is about 1/3 that of the basal part of the element. The spine curves slightly away from the other fingers, more strongly towards the tip, and extends into a feeler-like multi-annulated structure (flagellum) (Figure 7F, G). The basal part of the element contains a possible excretory gland (Figure 6D). Claw element 2 short, as wide (diameter) as long, straight, with an elongate, medio-distal spine, directed distally, 3x as long as the basal part of the element. The diameter of the spine at its base is about 1/3 that of the basal part of the element. The spine is curved slightly outward, more strongly towards the tip, and extends into a feeler-like multi-annulated structure (flagellum). Claw element 3 is almost half the diameter of the preceding element and only slightly shorter than the medio-distal spines of the preceding claw elements. It extends latero-distally into a feeler-like multi-annulated structure (flagellum; Figure 7D-F). Claw element 4 is a small hemispherical structure with four inwardly-curving (hook-like) spines differentiated in size (Figure 7A-C). The most distal spine (s1) is the largest (length about twice the width of element 4 at the base), the most proximal (s2) is the second largest (length similar to the width of element 4), the sub-terminal (s3) is the third largest (length about half the width of element 4), the second proximal spine (s4) is the smallest (length about one third the width of element 4).

#### Appendage 2

**Figure 8:**
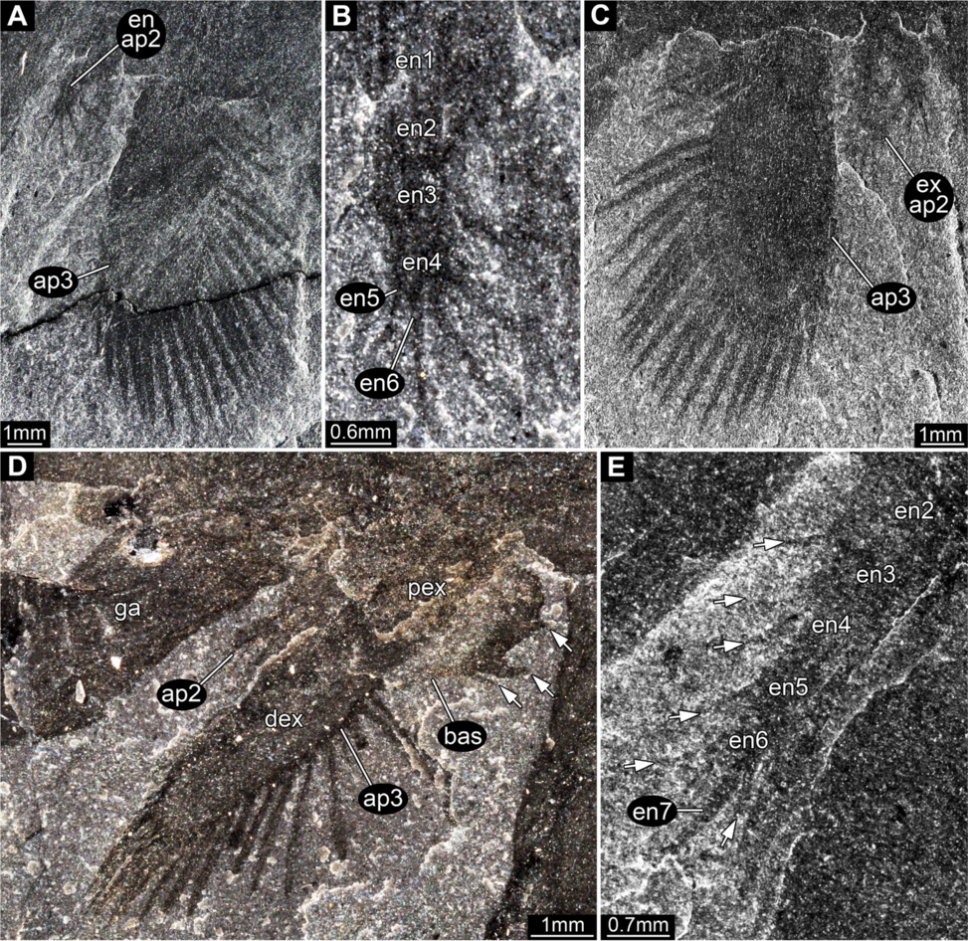
*** Leanchoilia superlata *Walcott, 1912, appendages two and three.A**- **C**, ROM 61880; **A**, part, note the very small size of appendage two compared to appendage three; **B**, Close up of appendage two; **C**, Counterpart; **D**, ROM 61895, lateral view of great appendage, appendage two and three (exopod bipartite), arrows mark basipod setation; **E**, ROM 61872, endopod of appendage three with setation (arrows); additional abbreviations: bas = basipod; dex = distal part of exopod; en = endopod; ex = exopod; pex = proximal part of exopod.

Significantly smaller than the more posterior appendages: only one third the length of appendage 3 (Figure 8A, C). Consists of basipod (?), endopod and exopod. The endopod consists of 6 elements (?) (Figure 8B). Elements 1 and 2 are longer than wide (diameter), unclear whether they are setose. Element 3 slightly longer than wide (diameter), with a long medio-distal seta. Element 4 as long as wide (diameter), with a long medio-distal seta. Element 5 very small, forming the base for element 6, with two long setae, one medio-distal, one latero-distal. Element 6 small, extending into an elongate distally pointing seta. Exopod paddle-shaped, about twice as long as wide, unclear whether bipartite. Exopod about 30% longer than endopod. Extrapolation based on the spacing of the approximately 5 preserved setae, indicates that there were orginally about 16.

#### Appendage 3

**Figure 9:**
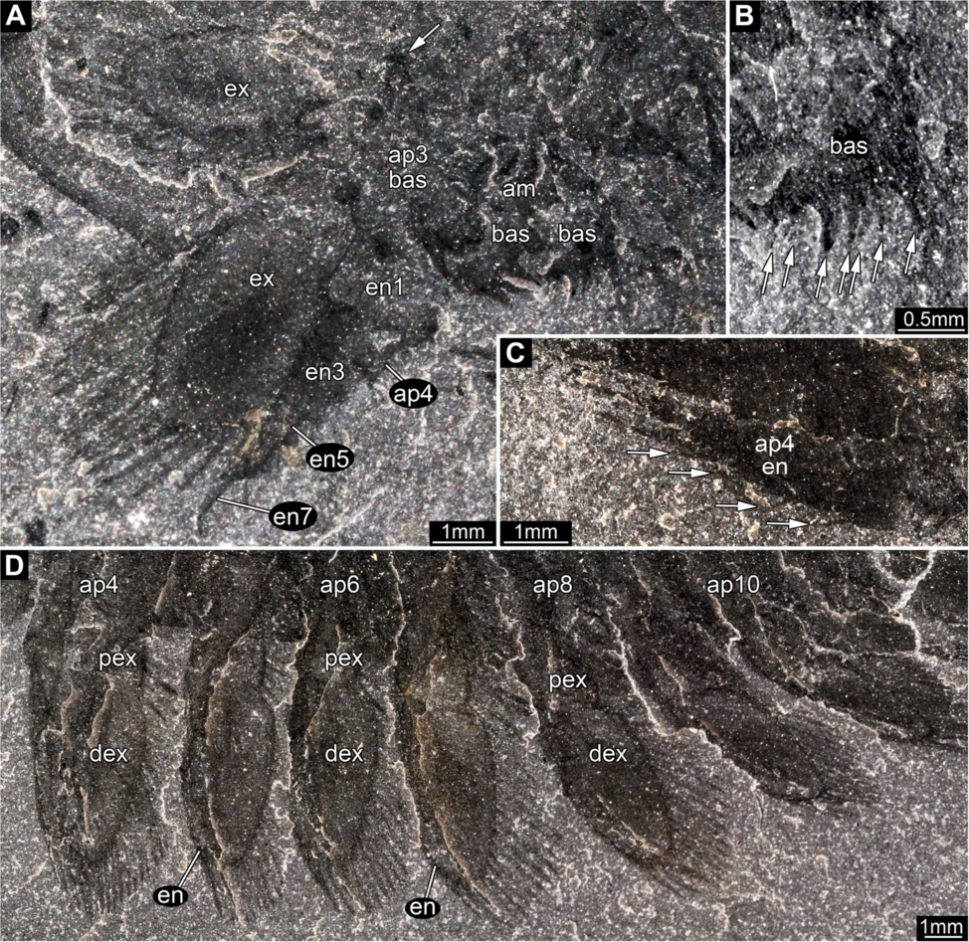
*** Leanchoilia superlata *Walcott, 1912, appendage three and more posterior appendages.A**- **B**, ROM 61901; **A**, Appendages three and four with preserved basipod, left and right basipod of appendage pair four in supposed life position, arrow indicates the far lateral position of the pivot joint; **B**, Close up of **A**, arrows mark setation; **C**, ROM 61907, endopod of appendage four with setation (arrows); **D**, ROM 61886, series of appendages; additional abbreviation: am = arthrodial membrane.

Significantly larger than appendage 2, length about two thirds that of the head shield. Basipod with 3 (?) groups of robust spines arranged from proximal to distal along the median edge (Figure 8D). Spine groups apparently triplets, probably with one central and two adjacent spines arranged in a line. Arthrodial membrane with three large folds occupies a medial notch in the basipod where it articulates with the body (Figure 9A). Endopod of 6 or 7 elements, their length similar to or slightly longer than width (Figure 8E), tapering progressively distally. One seta arises medio-distally from each element and an additional latero-distal seta is present on element 6. Element 7 about twice as long as element 6, length 5x width (diameter), curving slightly inwards. Exopod slightly longer than endopod (?), length about 2.5x width, bipartite, with a triangular proximal part and a paddle-shaped distal part (Figure 8D). Proximal part articulates with basipod and bears 3 spine-like setae laterally. Distal part articulates with endopod element 1 (?), and bears 16–18 spine-like setae around the margin (about 4 median, 1 distal, 11–13 lateral).

#### Appendage 4

Similar in morphology (Figure 9A) but larger than appendage 3, length similar to that of head shield. Basipod with 4 groups of robust spines arranged from proximal to distal along the median edge (Figure 9B). Spine groups apparently triplets, probably with one central and two adjacent spines. Basipod-body joint the same as that in appendage 3. Endopod consists of 7 elements, their length similar to or slightly longer than width (Figure 9A), tapering progressively distally. One seta arises medio-distally from each element (Figure 9C) and an additional latero-distal seta is present on element 6. Element 7 about twice as long as element 6, length 5x width (diameter), curving slightly inwards. Exopod slightly shorter than endopod, length about 2x width, bipartite, with a triangular proximal part and a paddle-shaped distal part (Figure 9D). Proximal part articulates with basipod and bears 4 spine-like setae laterally. Distal part articulates with endopod element 1 and preserves 16 spine-like setae of an estimated 19 (3 median, 1 distal, 12 (estimated 15) laterally).

#### Appendage 5

**Figure 10:**
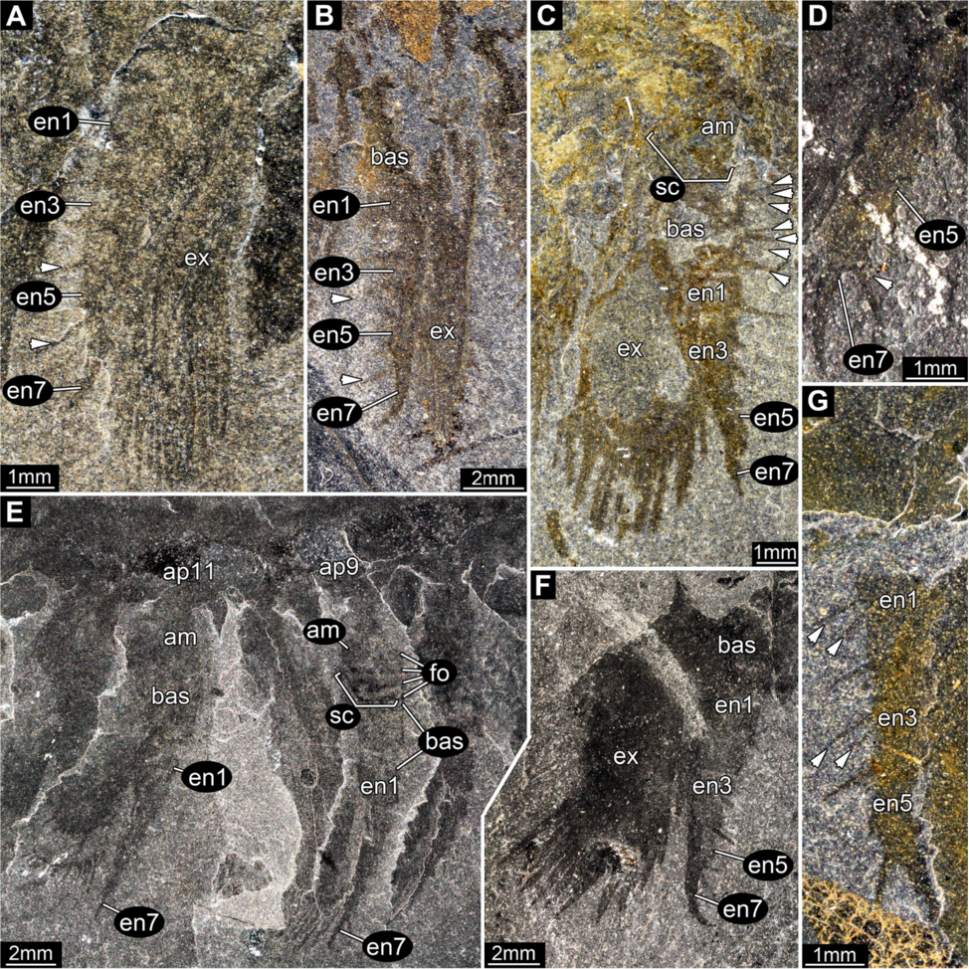
*** Leanchoilia superlata *Walcott, 1912, appendage five and further posterior appendages.A**, ROM 61903, appendage five; **B**, ROM 61865, appendage six; **C**- **D**, Appendage seven; **C**, ROM 61881, spine triplets apparent (arrows), note the curved sclerotic boundary between basipod and arthrodial membrane; **D**, ROM 61908; **E**, ROM 61879, appendages eight to twelve with pronounced folds of the arthrodial membrane and clearly evident sclerotic boundary on appendage nine; **F**, ROM 61904, appendage eight; **G**, ROM 61883, appendage twelve, only specimen with two setae on some endopod elements (arrows); arrows mark setation in all images; additional abbreviations: fo = folds; sc = sclerotic boundary (outline).

Similar in morphology (Figure 9D) but slightly larger than appendage 4, length similar to that of head shield. Neither basipod nor its body joint preserved but probably similar to those of appendage 4. Endopod consists of 7 elements, their length similar to or slightly longer than width (Figure 10A). Elements 1–6 tapering progressively distally. One seta arises medio-distally from each element and an additional latero-distal seta may be present on element 6. Element 7 about twice as long as element 6, length 5x width, curving slightly inwards. Exopod slightly shorter than endopod, length about 2x width, bipartite, with a triangular proximal part and a paddle-shaped distal part. Proximal part articulates with basipod and bears 4 spine-like setae laterally (Figure 9D). Distal part articulates with endopod element 1 (?) and bears 19 spine-like setae (estimated).

#### Appendage 6

Similar in morphology (Figure 9D) and size to appendage 5. Neither basipod armature nor body joint preserved, but presumably as in appendage 4. Endopod consists of 7 elements, their length similar to or slightly longer than width (Figure 10B). Elements 1–6 tapering progressively distally. One seta arises medio-distally from each element and an additional latero-distal seta may be present on element 6. Element 7 about twice as long as element 6, length 5x width, curving slightly inwards. Exopod slightly shorter than endopod, length about 2x width, bipartite, with a triangular proximal part and a paddle-shaped distal part. Proximal part articulates with basipod and bears 4 (?) spine-like setae laterally. Distal part articulates with endopod element 1 (?) and bears 19 spine-like setae (estimated).

#### Appendage 7

Similar in general aspect and size to appendage 6 (Figure 9D). Basipod with 3 or 4 groups of robust spines arranged from proximal to distal along the median edge. Spines grouped apparently as triplets, with one central and two adjacent spines (Figure 10C). Arthrodial membrane, possibly with three large folds, occupies a medial notch in the basipod where it articulates with the body (Figure 10C). Endopod consists of 7 elements (Figure 10C, D), 1–6 tapering progressively distally. All elements as long or slightly longer than wide. One seta arises medio-distally from each element and an additional latero-distal seta may be present on element 6. Element 7 about twice as long as endopod element 6, length 5x width, curving slightly inwards. Exopod slightly shorter than endopod, length about 2x width, bipartite with a triangular proximal part and a paddle-shaped distal part. Proximal part articulates with basipod and bears 4 (?) spine-like setae laterally. Distal part articulates with endopod element 1 and bears 16 spine-like setae (estimated 19).

#### Appendage 8

Similar in general aspect and size to appendage 7 (Figure 9D). Neither basipod armature nor body joint preserved, but presumably as in appendage 7. Endopod and exopod as in appendage 7 (Figure 10F). Distal part bears 19 spine-like setae.

#### Appendage 9

Similar in general aspect but slightly smaller than appendage 8; slightly shorter than the head shield. Basipod armature not preserved, presumably as in appendage 7. Basipod-body joint as in appendage 7 (Figure 10E). Endopod as in appendage 8. Exopod as in appendage 8 (Figure 9D), but proximal part bears only 3 (estimated) spine-like setae laterally. Distal part bears 17 spine-like setae (estimated 18).

#### Appendage 10

Similar in general aspect but slightly smaller than appendage 9. Neither basipod armature nor body joint preserved, but presumably as in appendages 7 and 9, respectively. Endopod details not preserved, presumably as in appendage 9. Exopod presumably as in appendage 9, but with 16 (estimated) spine-like setae (Figure 9D).

#### Appendage 11

Similar in general aspect but slightly smaller than appendage 10; length about four fifths of the head shield (Figures 9D, 10E). Neither basipod armature nor body joint preserved, but presumably as in appendages 7 and 9, respectively. Endopod as in appendage 8. Exopod presumably as in appendage 10.

#### Appendage 12

Similar in general aspect but slightly smaller than appendage 11; length about three quarters of the head shield. Neither basipod armature nor body joint preserved, but presumably as in appendages 7 and 9, respectively. Endopod as in appendage 8, but elements 1 and 3 have one adjacent seta each (Figure 10G). Exopod presumably as in appendage 10.

#### Appendages 13–15

Appendage 13–15 not preserved with details. Presumably similar in general aspect but slightly smaller than preceding appendages (Figure 2D).

## Discussion

Our description of *Leanchoilia superlata* differs significantly from earlier interpretations.

### Body shape

The dorsal morphology of both the head shield and tergites of *Leanchoilia superlata* was strongly trilobate. The body axis was slender and spindle-shaped (Figures 2B, E4A). The broad appearance of *L. superlata* in dorso-ventral view reflects the very wide tergo-pleurae relative to the axis (Figure 2B). García-Bellido and Collins [6], in contrast, interpreted the body of *L. superlata* as relatively broad and massive, extending across much of the width of the tergo-pleurae, which they depicted oriented ventrally ([6], their text-Figures nine and eleven A). Our observations show that the tergo-pleurae projected more or less horizontally (Figure 2D; [6], their Plate two, Figure one; [7], e.g., their Figures eighty-two, eighty-eight). This attitude allows the high lateral flexibility exhibited by various specimens of *L. superlata* (Figure 4A, C, D, H). Although dorsal flexure between tergites was limited by the backward projecting keels, specimens show that the animal could also curve to a large degree in this direction (either actively or passively) without disarticulating. *L. illecebrosa* from the lower Cambrian Chengjiang fauna, interpreted as sister species to *L. superlata,* has only been reconstructed in lateral aspect ([10], their Figure one). A pronounced trilobation with a slender axial region is evident, however, in well preserved specimens in dorsal view ([10], their Figure two F).

**Figure 11:**
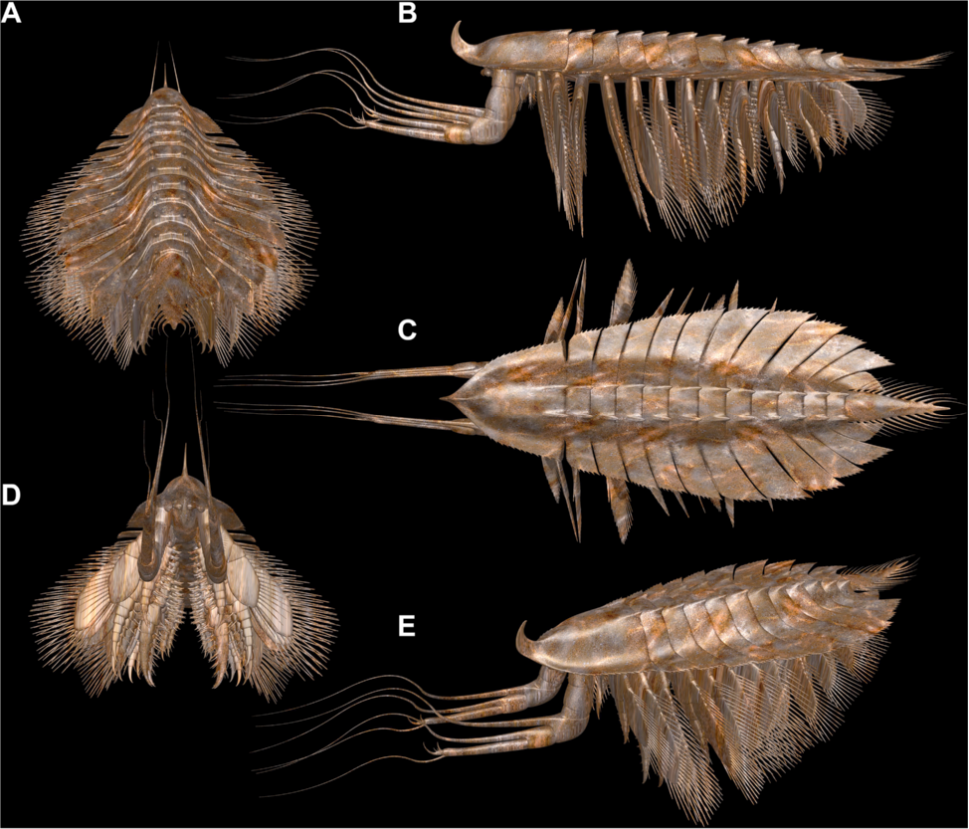
**Three-dimensional model of* Leanchoilia superlata *Walcott, 1912 illustrating new interpretation of the morphology.A**, Postero-dorsal view, note the position of the tergo-pleurae and the relatively narrow axial region; **B**, Lateral view, showing metachronal leg movement, note the slim body; **C**, Dorsal view; **D**, Antero-ventral view with strong median setation of appendages; **E**, Antero-dorso-lateral view; color of the model inspired by modern chelicerates.

The slenderness of the body is difficult to reconcile with the robust nature of the appendages when applying traditional 2D reconstruction tools. Only a three-dimensional reconstruction allows cross-referencing of laterally and dorso-ventrally preserved specimens, and it also facilitates a determination of the anterior aspect of the animal (Figure 11). García-Bellido and Collins [6] reconstructed a very massive body compared to our observations (see above), but their frontal reconstruction underestimates the relative size of the appendages (their text-Figure eleven A). The relative size of the appendages reconstructed by Bruton and Whittington [7] more closely matches our interpretation, no doubt aided by their three-dimensional model (made under Bruton’s direction), although they too overestimated the width of the body. Their reconstruction lacks proximal details of the appendages, which are revealed by new specimens and our photographic techniques.

### Eye structures

*Leanchoilia superlata* was originally described as possessing eyes [19,20]. Subsequent studies cast doubt on this interpretation and Bruton and Whittington [7] considered *L. superlata* to be blind. García-Bellido and Collins [6] provided clear evidence for the presence of eye structures in this species (for a more detailed history see [6], p. 698). They identified four eyes in total, interpreting them as “simple” due to the absence of evidence for ommatidia. Here we demonstrate that the structures on the left and right side of the head are connected and represent just two eyes, each with two lobes (Figure 3D, F). Similar structures in *L. illecebrosa*[10] were interpreted in the same way. The presence of a short stalk (Figure 3F) in *L. superlata* indicates that these are compound lateral eyes. The absence of evidence for ommatidia is presumably taphonomic; such evidence is unknown in Burgess Shale arthropods. The general organisation of the eye, e.g., bilobation, presence of a stalk, remains to be reinvestigated in the other leanchoiliid species.

We found no evidence to support the interpretation of the eye structures of *Leanchoilia superlata* and *L. illecebrosa* offered by Schoenemann [21]. She reported the presence of two sets of eyes, lateral (compound) eyes and median ocelli. The structures interpreted by Schoenemann [21] as ocelli in *L. superlata* are the compound eyes below the shield (Figure 3G, H). The structure that she interpreted as a stalked eye in lateral view in *L. illecebrosa* is part of the great appendage, as already shown by Hou and Bergström ([22], their Figure twenty-four). The phylogenetic position of *Leanchoilia* indicates that it possessed median eyes, but we could find no evidence of them on the fossils.

### Hypostome

A hypostome is a sclerotised plate covering the mouth opening. It is known especially in trilobites [23,24], other Burgess Shale arthropods [25], and also in early crustaceans [26,27]. While the hypostome appears among early sclerotised arthropods [28,29] it has not been reported previously in any short great-appendage arthropod [5]. The structure interpreted here as the hypostome in *Leanchoilia superlata* has the shape and position of that in other arthropods. Yet, compared to other known hypostomes of arthropods from the Burgess Shale it appears to have been less well-sclerotised [25]. The structure has been found in only a few specimens of *L. superlata*, but this is also true for other important details such as the short eye stalks (see above) and the tiny second appendage (see below).

### Great appendages

**Figure 12:**
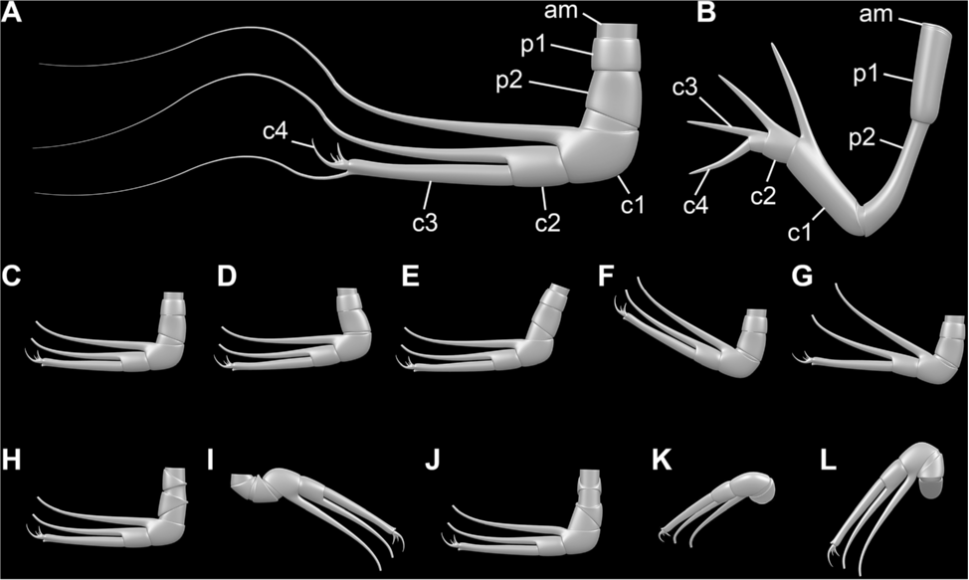
**Three-dimensional models of great appendages and their mechanics.A**, Great appendage of *Leanchoilia superlata* Walcott, 1912; **B**, Great appendage of *Yohoia tenuis* Walcott, 1912; **C**- **G**, Movability of the great appendage of *L. superlata* based on the new reconstruction herein; **H**- **L**, Rotation of the great appendage of *L. superlata* requiring deep notches in the peduncle, for which we could find no evidence; this range of movement may require disarticulation at the joints during preservation; abbreviations as before.

The morphology of the great appendages of leanchoiliid species has been interpreted in different ways (see Figure 12A for our interpretation). Some authors inferred the presence of two elements in the peduncle (e.g., [7], their Figure one hundred and eleven; [10,14,30,31]), but others favored a single element ([6], their text-Figure eleven A). Different interpretations of the nature of the multi-chela have also been offered. It has been interpreted as consisting of three [4,6] or four elements [10,14,30,31]. Interpretations involving four elements have proposed different configurations. Liu et al. [10] interpreted the distal flagellum as a fourth element, a possibility also discussed by Chen et al. [31]. Alternatively, Chen et al. [31] argued that a tiny claw segment (their Figure eight f, although they referred to their Figure eight g in the figure caption) could represent a fourth element. This last interpretation is supported by our reinvestigation. The distal armature of the great appendage does not consist of three simple spines arising from the third claw element as reconstructed by García-Bellido and Collins ([6], their text-Figure nine) and Bruton and Whittington ([7], their Figure one hundred and eleven). Instead, the distal armature arises from a movable fourth element (Figure 7A-C), which can become disarticulated (Figure 7D). Furthermore, this armature comprises four spines, differentiated in size. The largest of these distal spines is significantly larger than reconstructed by most authors ([6], their text-Figure nine). The extended spines of the more proximal elements are also longer than reconstructed before, distally opposing the spines of the fourth element, allowing an occlusion against the spine-bearing part. The great appendage appears to be a fully functional prehensile appendage, with the same number of elements as in other early megacheirans such as *Yohoia tenuis* or *Fortiforceps foliosa* Hou and Bergström, 1997 (Figure 12B; [5], their Figures five B, seven C)*.*

The great appendages can be preserved in different ways. In some specimens the fingers are broken off (Figure 6G), and the great appendage appears similar to that in *Yohoia tenuis*[5]. In others only the distal flagellum is missing (Figure 6E). The curvature of the two more proximal fingers varies from almost straight (Figure 6D) to more strongly curved (Figure 6E), but this variation may be preservational.

Another feature of the great appendage may also be an effect of preservation: its posteriorly directed position in some specimens. Bruton and Whittington ([7], p. 571) discussed whether this attitude (their Figures eighty-three, one hundred and seven) could represent a position in life and interpreted this as possible but not certain. They ([7], their Figure one hundred and twelve b) reconstructed the posteriorly directed position of the great appendage as an attitude during swimming, and this interpretation was accepted by later authors [6]. The difficulty with this interpretation is the movement required at the articulations (Figure 12C-G). The transition from projecting anteriorly to posteriorly demands either 1) a large membranous area, which would weaken the junction between the appendage and the body, or 2) a large notch in the most basal element of the great appendage. Evidence of such features has not been observed in any of the specimens investigated here. The two specimens used by Bruton and Whittington ([7], their Figures eighty-three, one hundred and seven) as a basis for their reconstruction appear to show a rotation of the appendages through 180°, based on the position of the spines. In order to achieve such a rotation without disarticulating the elements of the appendage, very extensive arthrodial membranes are required in the joints (Figure 12H-L) but there is no evidence for notches to accommodate them. The great appendages of *Leanchoilia superlata* that project posteriorly may have been subjected to some disarticulation (Figure 6F; [31]).

### The second appendage and the number of head appendages

García-Bellido and Collins [6] followed Bruton and Whittington [7] in reconstructing the head of *Leanchoilia superlata* as comprising three appendage-bearing segments, while *L. illecebrosa* (a supposed sister species) was reconstructed with four [10]. Edgecombe et al. [4] accepted this discrepancy as variation within the genus. We discovered that *L. superlata* possessed a very small second appendage, which has not been recognized before (Figures 3B8A-D). This appendage can be interpreted as a specialized mouth part. In addition to its significantly different size, it has much longer setae on the endopod than the succeeding appendages. Accordingly, *L. superlata*, like its sister species, has four appendage-bearing segments in the head. This feature requires reinvestigation in other leanchoiliids.

### Third and succeeding appendages

A rigid basipod with median spination was described in *Leanchoilia illecebrosa* and *L. persephone*[6,10], yet had not been reported previously in *L. superlata* (e.g., [6], their text-Figure eleven A). The apparently four spine groups observed here, at least on appendages 3–5 (Figure 9B; unclear on others), is an arrangement similar to the enditic structures on the basipod in *L. illecebrosa*[10]. But the armature in *L. illecebrosa* consists of single spines whereas there are spine triplets in *L. superlata* (Figure 10C). This feature requires reinvestigation in other leanchoiliids.

The endopod in *Leanchoilia superlata* never comprises more than seven elements. In *L. illecebrosa* it was reconstructed with nine elements [10]. García-Bellido and Collins ([6], their text-Figure six) interpreted the number of elements in *L. persephone* as nine, but they numbered their supposed most proximal element (the basipod) as endopod element 1 (“bearing gnathobases”), and did not number the distal claw. A comparison of the specimen they refer to in this context ([6], their text-Figure six) with similar specimens of *L. superlata* (Figure 10C) suggests that their “podomere 3” corresponds to the basipod. If this interpretation is correct, the structure of the endopod in *L. persephone* is similar to that in *L. superlata*. It is clear that an endopod with nine elements is not necessarily a ground pattern feature of leanchoiliids, and may not be a reliable basis for excluding the group from Euarthropoda (cf. [16], character 30 of their phylogenetic analysis).

While the endopods of most appendages of *Leanchoilia superlata* bear one seta per element, we discovered one specimen that preserves the endopod of appendage 12 and it shows two setae on elements 1 and 3 (Figure 10G). The setae on the endopods of the preceding and succeeding appendages of this specimen are not preserved so it is not known whether this pattern is unique to appendage 12 or to this specimen. With this uncertainty, we did not include this setation pattern in the reconstruction. The setation pattern requires reinvestigation in other leanchoiliids.

The general appearance of the exopods of *Leanchoilia superlata* has often been reconstructed as feather-like ([6], their text-Figure nine; [7], their Figure one hundred and eleven b), as if the outer rim was deeply divided. Additionally, the supposed filaments resulting from this division were reconstructed with a rounded tip. We could not confirm this interpretation. The exopod paddle has a well defined rim (Figures 9A, C, D; also [6], their Plate two, Figure two), from which elongate pointed structures arise. There is no evidence that these structures are filamentous and we interpret them as setae.

### Functional morphology of the biramous appendages

The exopod of the third and succeeding appendages has been described as comprising two elements in *Leanchoilia superlataL. persephone* and *L. illecebrosa*[6,10]. Yet, the nature of the articulation has been reconstructed only in *L. illecebrosa* and its functional morphology discussed only briefly [10]. The main function and the evolutionary novelty represented by this articulation have not been recognized, although it represents a specialized bio-mechanical system. This system, with the proximal part of the exopod articulating with the basipod and the distal part of the exopod articulating with endopod element 1, occurs in appendage three and those following, and probably also in appendage two, although details of the last are not available due to its small size.

**Figure 13:**
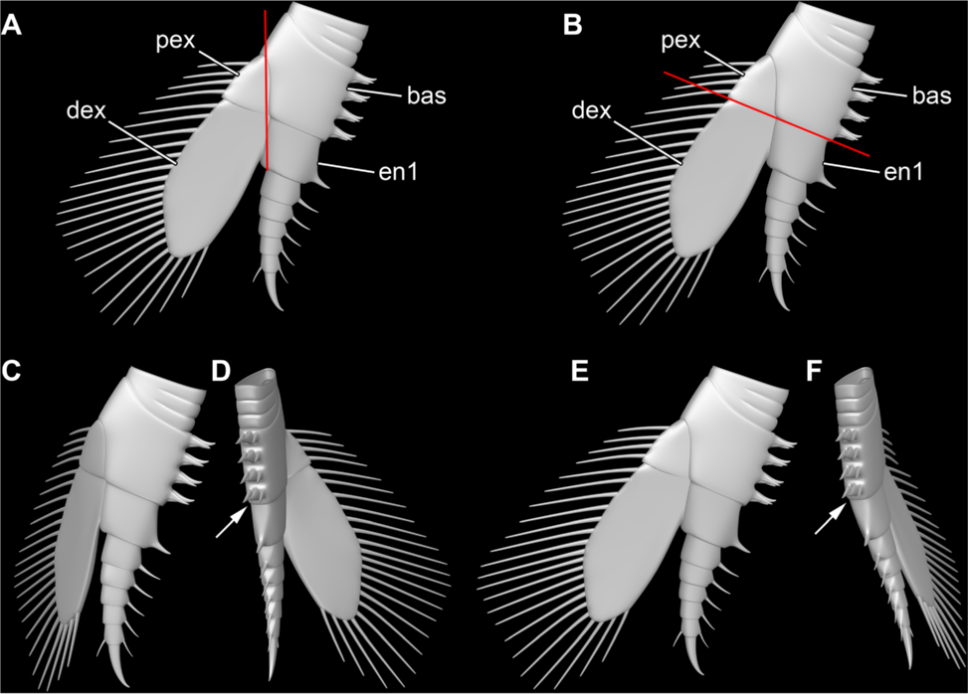
*** Leanchoilia superlata *Walcott, 1912, functional morphology of the biramous appendages.A**- **B**, Joint systems; **A**, Red line indicates joint of proximal part of exopod (pex) + distal part of exopod (dex) *vs* basipod (bas) + endopod element 1 (en1); **B**, Red line marks joint of proximal part of exopod + basipod *vs* distal part of exopod + endopod element 1; **C**- **D**, Recovery stroke, exopod moving posteriorly as joint pex + dex *vs* bas + en1 is bent; **C**, Anterior view; **D**, Median view, basipod and endopod element 1 in line (arrow); **E**- **F**, Power stroke, exopod movement enabled as joint pex + bas *vs* dex + en1 is bent; **E**, Anterior view; **F**, Median view, basipod bent against endopod element 1 (arrow).

The oblique articulation between the exopod and the basipod and endopod element 1 allows the exopod to swing posteriorly thereby reducing drag during the recovery stroke (Figure 13A). Yet, the same articulation would reduce the effectiveness of the power stroke if the exopod could swing anteriorly. The transverse articulation between the basipod and proximal exopod and the rest of the limb prevents an anterior swing (Figure 13B). The exopod can only swing posteriorly or anteriorly when endopod element 1 is aligned with the basipod (Figure 13C, D). As soon as the limb is flexed at the transverse articulation the exopod is locked and cannot swing anteriorly; this position was presumably used in the power stroke (Figure 13E, F). This is a more evolved system than the normal reliance on flexure during the recovery stroke. Our interpretation, which is based on the construction of a three-dimensional model, renders the combined flexure of endopod and exopod illustrated by Liu et al. ([10], their Figure six D) impossible.

The extensive arthrodial membrane at the limb–body junction shows four distinct folds on the inner margin. The limb appears to articulate with the body (pivot joint) on the outer margin; thus the arthrodial membrane allowed it to swing outwards during the power stroke. Liu et al. [10] also suggested a degree of rotation (their Figure six D). Inward and outward movement of the appendages probably also functioned in feeding, providing the possibility of crushing food between the basipod spines. Such a movement is reminiscent of that used by different types of mandibles ([32], their Figure two.four.six).

### Consequences

The new understanding of the morphology of *Leanchoilia superlata* prompts a re-evaluation of its autecology. The slender body and relatively large limbs, together with the presence of stalked compound eyes, point to a more active mode of life than previously inferred. The endopods do not have a tip suitable for walking, and the long exopod setae extend distally beyond the tip of the endopods. These observations, together with the sophisticated exopod articulation and the flexibility of the limb–body junction, indicate that *L. superlata* was a swimmer.

There is no evidence that the exopods functioned as gills (e.g., [33]; see [34] for an extensive discussion). Gills are specialized organs with a particular ultrastructure that facilitates gas exchange. This cannot be inferred for the fossils. The large number of setae on the exopods enlarged the body surface and presumably enhanced gas exchange. Yet, their primary function appears to be for swimming. Similar considerations apply to all Burgess Shale arthropods; no exopod in any extant taxon is just a gill, but exopods may carry gill structures (e.g., in Xiphosura, Stomatopoda).

The armature of the appendages of *Leanchoilia superlata* indicates a predatory lifestyle. Both the basipods and endopods are equipped with spines and are unlikely to have been used for walking (supporting [7], p. 576). The specialized basal articulation that facilitates swimming is also advantageous for processing food. Such articulations, with a far laterally positioned pivot joint close to the body proper and a larger membrane on the median side, are often associated with mandibles, indicating that *L. superlata* could crush food items between its basipods. Most obviously the multi-chela of the great appendage, with its distal feelers, is a raptorial structure. Butterfield [35] argued that the phosphatic gut fill indicates a predatory (or scavenging) mode of life. *L. superlata* appears to have been an agile necto-benthic predator, actively pursuing prey.

Accordingly, *Leanchoilia superlata* is not interpreted as a scavenger or mud eater (contra [7,36]). Possible prey items are more difficult to identify. The prey of *Yohoia tenuis* has been supposed to include the smallest arthropods such as agnostines and bradoriids [5]. Based on its size, *L. superlata* probably fed on larger prey items. However, there is no evidence that it fed on scalidophoran worms like *Ottoia* as suggested for *L. illecebrosa* ([31], their Figure twelve). Specimens of *Ottoia prolifica* Walcott, 1911 often occur on the same slab as specimens of *L. superlata*. These specimens of *O. prolifica* are about the same size as the specimens of *L. superlata* and it is more likely that both predatory species fed on the same type of prey rather than on each other.

## Conclusions

Our interpretation of the morphology and autecology of *Leanchoilia superlata* differs in important respects from earlier versions. Our study, like some other recent reinvestigations [5,25,37] took advantage of new investigation techniques (polarized light, composite imaging) and the availability of more taxa for comparison. Paleoecological reconstructions of the Burgess Shale biota depend on autecological interpretations of individual organisms and will be refined by future reinvestigations of other organisms.

Similarly, current phylogenetic analyses of the Burgess Shale arthropods will be improved by new morphological investigations (for a more extensive discussion, see [5,25]). Future application of a descriptive matrix approach such as that used here will facilitate comparisons between taxa and the construction of new data matrices.

## Authors’ contributions

JTH and CH documented the specimens, processed the images and created the descriptive matrix. JTH created the 3D computer models and measured the specimens. All authors participated in the morphological interpretations and writing of the manuscript, and read and approved the final manuscript.

## Supplementary Material

Additional file 1 **Descriptive matrix of* Leanchoilia superlata *Walcott, 1912 used for this re-description.** (XLS 38 kb) Click here for file
